# Changes in Fat Oxidation and Body Composition after Combined Exercise Intervention in Sedentary Obese Chinese Adults

**DOI:** 10.3390/jcm11041086

**Published:** 2022-02-18

**Authors:** Jingguo Cao, Siman Lei, Tong Zhao, Yuting Xie, Zunqiang Zhou, Sulin Cheng, Xiuqiang Wang

**Affiliations:** 1 Exercise, Health and Technology Centre, Department of Physical Education, Shanghai Jiao Tong University, Shanghai 200240, China; 370321199703212716@sjtu.edu.cn (J.C.); alicelei@um.edu.mo (S.L.); zt735702688@sjtu.edu.cn (T.Z.); xyt121251910012@sjtu.edu.cn (Y.X.); shulin.cheng@jyu.fi (S.C.); 2 Faculty of Education, University of Macau, Avenida da Universidade, Taipa, Macau 999078, China; 3 Sixth People’s Hospital Affiliated to Shanghai Jiao Tong University, Shanghai 200030, China; surgeon_zhou@163.com; 4 Faculty of Sport and Health Science, University of Jyväskylä, 40014 Jyväskylä, Finland; 5 Exercise Translational Medicine Centre, Shanghai Centre for Systems Biomedicine, Shanghai Jiao Tong University, Shanghai 200240, China

**Keywords:** obesity, exercise, fat loss, fat oxidation at rest, maximal fat oxidation

## Abstract

(1) Background: Evidence suggests that aerobic exercise and high-intensity interval training (HIIT) might increase fat oxidation and reduce fat. However, limited research has examined the effects of combining progressive aerobic exercise and HIIT interventions in sedentary adults with overweight and obesity, and differences in its effects between men and women remain unclear. The purpose of this study was to investigate the effects of combined progressive aerobic exercise and HIIT (CAEH) on fat oxidation and fat reduction in sedentary Chinese adults and compare sex differences in sedentary adults after seven weeks. (2) Methods: Eighty-four sedentary obese adults were enrolled and allocated to two groups in baseline (experimental (EXP) group:42; control (CON) group:42), and fifty-six subjects (EXP:31; CON:25) completed the experiments and were included in the final analysis. Subjects in the EXP group performed CAEH three times per week for seven weeks. Subjects in the CON group were advised to continue with their normal daily activities. Anthropometric, lipid profile, cardiorespiratory fitness, and fat oxidation outcomes were assessed before and after the intervention. (3) Results: After seven weeks of the CAEH intervention, compared with the CON group, the EXP group showed significant increases in fat oxidation at rest (FO_rest) (+0.03 g/min, *p* < 0.01), maximal fat oxidation (MFO) (+0.05 g/min, *p* < 0.01), and maximal oxygen intake (VO_2_max) (+3.2 mL/kg/min, *p* < 0.01). The changes in the percentages of the FO_rest (+57%) and the VO_2_max (+16%) were significantly greater (+20%, +6%) in males than in females (*p* < 0.05, *p* < 0.05). The body mass index (BMI) (−1.2 kg/m^2^, *p* < 0.01), body fat percentage (−3.2%, *p* < 0.001), visceral fat area (−12.8 cm^2^, *p* < 0.001), and total cholesterol (TC) levels (−0.4 mmol/L, *p* < 0.05) were significantly decreased in the EXP group. (4) Conclusions: Seven weeks of the CAEH intervention effectively improved FO_rest, MFO, and VO_2_max in sedentary obese adults, and the improvements in FO_rest and VO_2_max were more pronounced in males than in females. CAEH also improved body composition and TC levels in sedentary obese adults.

## 1. Introduction

Insufficiently active people have a 20–30% increased risk of mortality [1]. Epidemiological studies from China and other countries have observed positive relationships among sedentary behaviors (SBs) associated with an increased risk of obesity [2]. A study that was focused on Chinese adults showed that some of the metabolic markers also potentially attributed approximately 50% of the harmful effects of a sedentary lifestyle on occlusive cardiovascular disease (CVD) [3]. In addition, Chinese adults with over eight hours per day of SB are defined as having longer periods of SB, which is considerably associated with a higher risk of premature mortality [4]. Previous meta-analyses showed a non-linear association between sedentary behavior and risk of overweight/obesity by analyzing 21 of 58 studies published up to 2019 [5]. Moreover, Pan et al. summarized major national surveys of the past few decades, and pointed out that Chinese adults have reduced their total volume of physical activity, leading to an increased risk of obesity [6]. The Obesity Medicine Association has pointed out that the percent of body fat assessed by various validated machines is more accurate in defining obesity and has set the cut-off points for at >25% for males and >30% for females [7].

Therefore, it is essential to help reduce the detrimental effects of SB and develop strategies for fat loss and reversing obesity. Growing evidence suggests that fuel oxidation is a potential factor that might be beneficial for reducing body fat in sedentary obese people [8]. In addition, studies have shown that losing excess body fat is difficult in obese sedentary people, and that this may be caused by fat metabolism impairment and low-fat oxidation [9,10]. It has long been known that the oxidation rates of carbohydrates and fats are significantly lower, resulting in a decrease in fat utilization in obese people [11]. In addition, evidence has indicated that regular structured exercise can effectively prevent rebound fat gain, while also improving cardiorespiratory endurance and fat oxidation capacity, as well as enhancing metabolic flexibility [12].

Furthermore, studies have found that seven weeks of exercise intervention can significantly reduce fat and increase the fat oxidation rate [13]. In addition, studies have found that the maximum fat oxidation intensity of the human body during exercise primarily occurs within an exercise intensity range of 45–75% VO_2_max [14,15,16]. Exercise intensity directly affects the transfer of fatty acids and changes in corresponding hormones, which in turn affects the fat oxidation rate [17,18]. Furthermore, Lazzer et al. found that the fat oxidation rate increased significantly between the 50–70% VO_2_peak exercise intensity [19]. A recent meta-analysis summarized 11 trials and concluded that FM training improved maximal oxygen consumption and reduced fat mass at short- and medium-term time periods (8–20 weeks) among obese adults [20].

Additionally, lipid oxidation is also altered by factors such as training status, exercise intensity, duration, and sex [14]. Numao et al. [21] investigated the relationship between the oxidation of substrates between genders during exercise. They controlled the exercise intensity of obese men and postmenopausal obese women under 50%. According to observations by Knechtle et al. [22], for people under a steady exercise intensity when compared with men, women had a larger fat energy supply ratio. However, few studies have investigated the effects of fat oxidation from exercise between men and women in a trial.

Different exercise training programs are formulated according to the change law of fat oxidation during exercise. Exercise increases a body’s energy consumption and oxidizes and decomposes excess fat in the body. There is evidence that high-intensity interval training (HIIT) may cause more abdominal fat loss [23]. Specifically, fat decomposition in adipose tissue and muscle and the oxidation of fatty acids in muscle is accelerated [24]. Current exercise guidelines recommend medium-intensity continuous training for fat loss, primarily because it can increase energy consumption through long-term exercise and promote fat oxidation [19]. Studies have found that exercise can improve the body’s fat oxidation ability [12]. Continuous aerobic exercise takes a long time and people can easily feel that it is monotonous, but incremental-load aerobic exercise and interval training have varying loads, and both exercise methods improve fat oxidation and reduce fat [25,26]. Therefore, it can be assumed that a combination of progressive aerobic exercise and HIIT may be more suitable for people who lack exercise experience.

The new guideline developed by the World Health Organization (WHO) to promote physical activity (PA) and reduce SB suggested that people begin habitual activity gradually and arrange for more than 150 min that includes moderate to vigorous-intensity PA per week [27]. Combined progressive aerobic exercise and HIIT (CAEH) sessions may benefit fat oxidation (FO) and the endurance capacity that contribute to body fat loss maintenance [28]. In addition, the variety of this prescription may increase motivation and result in meeting PA guidelines.

It is necessary to apply a short intervention time to achieve possible improvements in body composition and fat oxidation capacity to encourage sedentary adults to maintain a healthy weight for a long time [29]. Recently, Chávez-Guevara et al. [20] reported in their meta-analysis that short- and medium-term durations might reduce body weight and body fat (BF), increasing cardiovascular fitness in low physical fitness individuals with obesity. However, to the best of our knowledge, the effects of combining HIIT with progressive intensity aerobic exercise have not been examined in sedentary obesity. Therefore, the purpose of the present study is to investigate the effects of CAEH on body composition, lipid profile, fat oxidation, and the cardiorespiratory fitness in sedentary obese adults. It is hypothesized that CAEH will improve measured variables in the participants in the exercise group (experiment) compared with the non-exercise (control) group.

## 2. Materials and Methods

### 2.1. Participants

This study was a quasi-experimental (non-randomized) controlled trial. Sedentary obese adults were recruited from the Shanghai Jiao Tong University using workshops, leaflets, social media, official web pages, and paper bulletins on campus.

**Figure 1:**
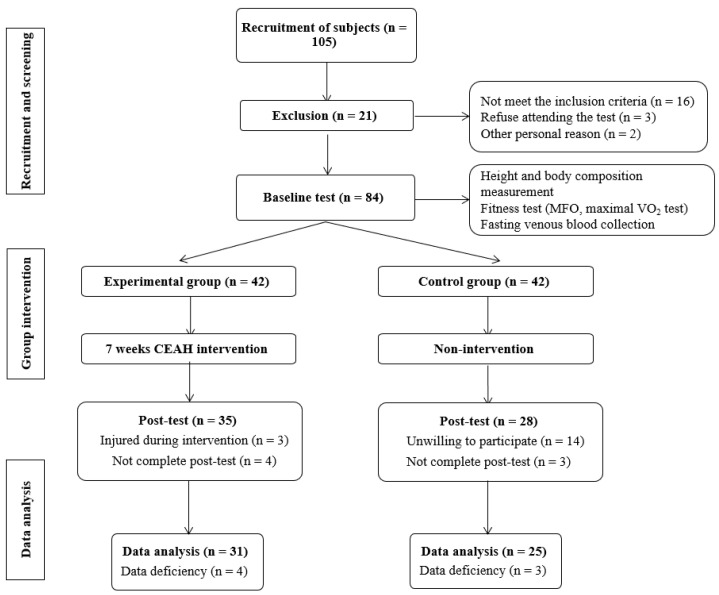
The flow chart of the experiment.

The study was part of a project called the Construction and Application of Health Management Model for Sedentary Obese registered at the Chinese Clinical Trials Register website (http://www.chictr.org.cn/index.aspx) accessed on 9 June 2021 (Number: ChiCTR2100047173), and the study adhered to the Principles of the Helsinki Declaration and the Data Protection Act. The ethics committee of Shanghai Jiao Tong University approved all interventions. A total of 105 respondents were invited to visit the research center. Prior to collecting eligibility data, researchers carefully explained the purpose and study protocol in detail. Prior to the registration, the participants signed a written informed consent letter. Five respondents refused to enroll in the study, and 100 participants completed an initial screening by filling out questionnaires on background information (including brief sedentary questions, medical history, current health status, American College of Sports Medicine (ACSM), a three-day (two weekdays and one weekend day) dietary record, an IPAQ-SF [30], and a physical examination. Sixteen did not meet the inclusion criteria of sedentary obesity, and a total of 84 participants were invited to enroll in this study. The experimental process is shown in Figure 1.

### 2.2. Inclusion and Exclusion Criteria

The inclusion criteria of the subjects in this study included: (1) being apparently sedentary according to living a sedentary lifestyle (spending more than eight hours awake in a sitting position daily and continuously sitting still for over 90 min); (2) age between 18 and 65; (3) a body fat percentage: male > 25%; women > 30%; (4) not engaged in regular exercise for three to five months, or in one year, for less than 30 min or less than two times a week; and (5) IPAQ (<600 metabolic equivalents (MET)·min/week).

The exclusion criteria included: (1) a body mass index (BMI) > 40; (2) severe cardiovascular or skeletal muscle disease; (3) serious complications of diabetes; (4) mental illness; (5) patients with other sports contraindications; or (6) pregnant and breastfeeding women.

The included subjects were divided into groups. Participants who were willing to engage in a weekly exercise program were included in the experimental (EXP) group (n = 42), and those who were unwilling to work out were included in the control (CON) group (n = 42). A total of 21 subjects (seven in the EXP group and 14 in the CON group) were either injured (not due to CAEH intervention) during the intervention, did not complete the post test, or were unwilling to participate. In addition, seven subjects were excluded due to severe data deficiencies. A total of 56 subjects were included in the final statistical analysis: the EXP group (n = 31) and CON group (n = 25).

### 2.3. Anthropometrical and Body Composition Assessments

Body height (cm) was measured using a stadiometer with standardized protocols. Bodyweight and composition were assessed using multi-frequency whole-body bioelectrical impedance analysis with an InBody 720 (Biospace Co., Ltd., Seoul, Korea). Subjects avoided exercising and caffeine, did not shower before testing, fasted overnight and arrived at the lab in the morning. They removed all metal objects from their bodies, and had to stand barefoot (standard mode) on an Inbody 720. Next, researchers gathered information about the subjects (age, sex, and height). The weight value was determined by the thumbs and four fingers of both hands touching the bottom electrode handle, and then the arms were dropped naturally. The torso was 15 degrees, facing forward, and the position was held until the end of taking the measurements (a total of 90 s). Speak or moving was not permitted during the measurements.

Body mass index (BMI) was calculated as body mass divided by the square of the body height (kg·m^−2^).

### 2.4. Lipid Profile (LP)

Venous blood samples (20 mL) were drawn from the antecubital vein in the morning after 12 h of overnight fasting. Serum was separated within 30 min and stored at −80 °C until analyses. Total cholesterol (TC), high-density lipoprotein cholesterol (HDL-C), low-density lipoprotein cholesterol (LDL-C), and triglycerides were analyzed using the enzymatic, colorimetric method with an automatic biochemical analyzer (Mindray BS-220, Shenzhen, China).

### 2.5. Exercise Test

#### 2.5.1. Maximal Graded Exercise Test (GXT)

A power bicycle (MONARK LC7) was used to test the maximum fat oxidation. The test scheme was an incremental load test. Subjects were tested with 50 W as the initial exercise intensity, and the load was increased every three minutes, 20 W each time, and the subject’s riding speed was ≥60 RPM. The exercise continued until the subject was exhausted or unable to maintain 60 RPM. The recovery load after exercise was 30 W and lasted for five minutes. At the beginning of the test, and the end of each exercise period, subjective fatigue rating of perceived exertion (RPE) as well as heart rate were recorded. Fingertip blood glucose and blood lactic acid were measured at the beginning, end, and at intervals during the exercise to avoid the risk of hypoglycemia. Subjects wore a CORTEX Portable Respiratory Metabolism Monitor and a Polar H10 heart rate band at all times.

The test was stopped immediately if the subject showed symptoms of dizziness, nausea, or dyspnea during the test.

The VO_2_ and VCO_2_ data for 30 s of each exercise phase, before the respiratory quotient, was greater than that selected and substituted into the following equation to calculate the fat oxidation rate: Fat Oxidation Rate (g/min) = 1.67 ∗ VO_2_(L/min) − 1.67 ∗ VCO_2_ (L/min). Then, the quiet oxidation rate (FO_rest) and maximum oxidation rate (MFO) of each subject were selected. The exercise intensity corresponding to the MFO was the maximum fat oxidation intensity (FATmax).

#### 2.5.2. Rowing Test

The maximum rowing machine load test method is described as follows. A 1 kg weight was used as the initial load intensity, and this was increased by 1 kg every two minutes. The rowing machine frequency was 26 times/min, and the rowing frequency lasted until the subject was exhausted or unable to maintain 26 times/min. The land rowing machine was only used for the CAEH, but not for the capability testing and data collection.

### 2.6. CAEH Intervention

According to the training program, subjects underwent CAEH intervention for seven weeks (three to nine weeks). The exercise program consisted of three sessions: preparation, main exercise, and cool-down.

The preparation session was approximately five minutes of freehand exercises (4 × 8 beat). The main exercise session lasted approximately 40 min, and each subject had a personalized training program (training sheet). Training consisted in three parts. The first part of the main exercise session was a five-minute warm-up with the land rowing machine with the minimum load, and the rowing frequency was 26 times per minute. The second part was the power cycling exercise with an increasing load. The exercise intensities were 40%, 50%, 60%, 65%, and 70% of the maximum load. Each exercise load lasted for three minutes for a total of 15 min. The third part was HIIT, which was performed on the land rowing machine. Three minutes of exercise at 40% maximum load, followed by one minute at 90% maximum load, five cycles, and the total time of the HIIT was 20 min. Finally, the session of cool-down involved approximately five minutes of static stretching, each stretch lasting 20 to 30 s, for a total of five minutes. One week prior to beginning the intervention, subjects were invited to join a pre-intervention workshop and were instructed on how to familiarize themselves with the movement and speed of rowing and the cycle ergometer, as they were unfamiliar with exercise training. Instructors guided and used heart rate monitors during exercise, and subjects controlled the intensity and recorded the RPE and heart rate for each session and for each part. In addition, researchers reviewed the frequency of attendance, weekly, and reminded them to participate.

### 2.7. Dietary Recommendation

Participants were asked to follow their individualized dietary recommendation during the study; however, diet was not assessed as part of this study.

### 2.8. Statistical Analyses

This study was designed as a non-randomized control trial. For estimating the sample size, the statistical program G-power 3.19 was utilized; the large effect size (ES = 0.8) was set based on Cohen’s d, the significance level was set to α = 0.05, and the power was (1 − β) = 0.8. This resulted in a minimum 26 of participants; however, a drop-out rate of 20% was considered, thereby setting the minimum number of participants to 33.

All of the data in this study were collected longitudinally and were measured twice from baseline to the end of the intervention. The experimental data were processed using Jamovi (2.0) statistical software and were presented as mean values and standard deviations (mean ± SD). A parametric test was used for data conforming to a normal distribution, and a non-parametric test was used for data not conforming to a normal distribution. Prior to variance analyses, data that did not conform to the normal distribution were transformed.

A paired sample T-test was used to analyze the indexes of all the subjects, before and after the intervention, and the indexes of the fat oxidation capacity and maximal oxygen uptake of males and females in the EXP group before and after the intervention. An independent sample T-test was used to test the differences in the baseline indexes between the EXP and CON groups and to compare the percentage change in the fat oxidation capacity and the maximal oxygen uptake in the EXP group between males and females. A repeated measures ANOVA was used to compare the time difference in the intervention effect on the evaluation variables. A *p*-value less than 0.05 represented significance in this study.

## 3. Results

### 3.1. Baseline Characteristics

The baseline characteristics are summarized in Table 1. A total of 84 subjects with excess body fat were enrolled in the study (105 were initially recruited), and the drop-out rate throughout the intervention period was 33%. A total of 56 subjects completed all of the interventions. A total of 24 of them were male (body fat percentage: 29.5 ± 4.4%) and 32 were female (body fat percentage: 34.9 ± 3.8%). There were no significant differences in the baseline information, fat oxidation capacity, body composition, and lipids profiles between the CON and EXP groups (Table 1).

### 3.2. Effect of Training on the Body Compositions and Lipid Profiels

After seven weeks of the CAEH intervention, the BMI of the EXP group decreased significantly (−1.2 kg/m^2^, *p* = 0.009, ES = 0.487), and there was an interaction effect between exercise and the intervention time (F = 4.17, *p* = 0.046, *η*^2^ = 0.008). The body fat percentage in the EXP group decreased significantly (−3.2%, *p* < 0.001, ES = 1.077), and there was a primary effect from the intervention time (F = 24.436, *p* < 0.001, *η*^2^ < 0.001). The VFA in the EXP group decreased significantly (−12.8 cm^2^, *p* < 0.001, ES = 0.973), and there was a primary effect in terms of the intervention time (F = 21.87, *p* < 0.001, *η*^2^ < 0.001). There were no significant changes in skeletal muscle in both groups, pre- and post-intervention. The TC level in the EXP group was decreased significantly (−0.4 mmol/L, *p* = 0.009, ES = 0.482), while the TC in the CON group had no significant change, and there was a major effect related to the intervention time (F = 4.247, *p* = 0.046, *η*^2^ = 0.046). There were no significant changes in the TG, HDL-C, and LDL-C levels in both groups, before and after intervention (Table 2). The ∆ (body fat percent), ∆ (VFA) and ∆ (TC) of the EXP group were significantly different from the CON group.

### 3.3. Effect of Training on Fat Oxidation and VO_2_max

**Figure 2:**
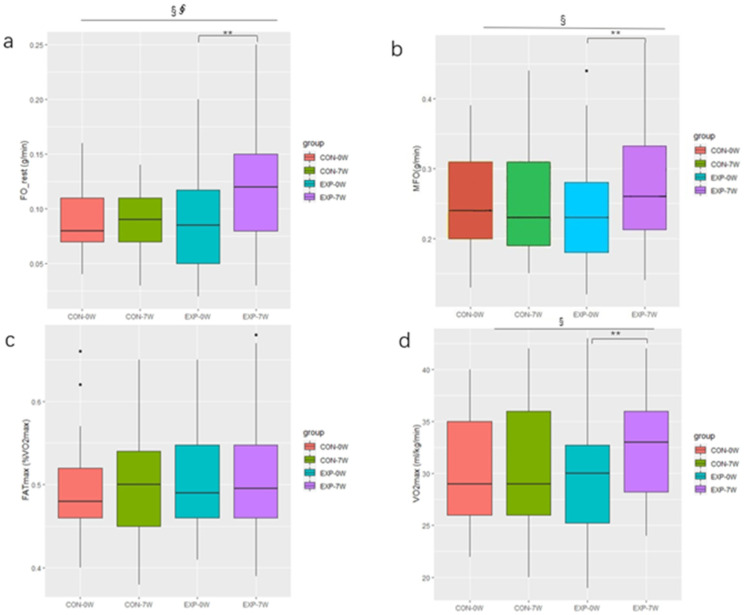
Change in fat oxidation and VO_2_max, pre- and post-intervention. ** denotes a significant difference *(p <* 0.01), ^§^ indicates that there was a primary effect from the intervention time (*p* < 0.05), ^∮^ indicates the interaction effect between the CAEH and the intervention time (*p* < 0.05), CON denotes the control group, and EXP denotes the experimental group, a denotes FO_rest of subjects pre- and post-intervention, b denotes MFO of subjects pre- and post-intervention, c denotes FATmax of sujects pre- and post-intervention, d denotes VO_2_max of subjects pre- and post-intervention.

**Figure 3:**
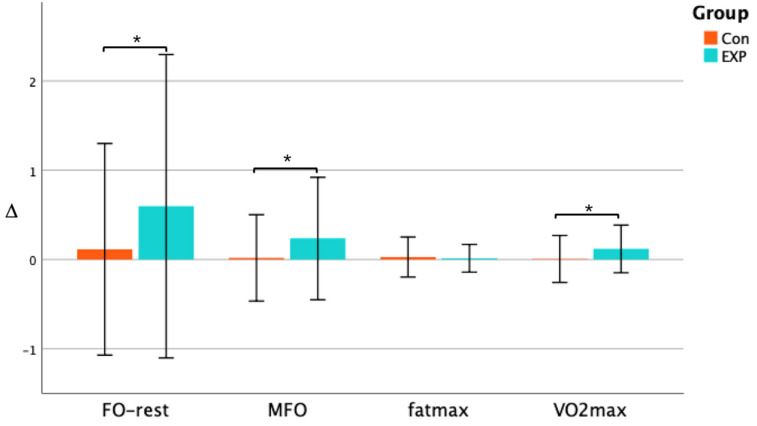
Change in fat oxidation and VO_2_max of in the CON group and EXP group. * denotes a significant difference (*p* < 0.05), and ∆ denotes the change pre- and post-intervention.

Prior to the intervention, there was no significant difference in the fat oxidation capacity and VO_2_max between the EXP group and the CON group. After seven weeks of the CAEH intervention, the FO_rest increased significantly (+0.03 g/min, *p* < 0.01, ES = 0.637) in the EXP group, and there was a primary effect of intervention time (F = 4.16, *p* = 0.047, *η*^2^ = 0.027), as well as an interaction effect of CAEH and intervention time (F = 4.83, *p* = 0.033, *η*^2^ = 0.031) (Figure 2a). The MFO increased significantly (+0.05 g/min, *p* = 0.002, ES = 0.636) in the EXP group, and there was a primary effect of intervention time (F = 4.29, *p* = 0.044, *η*^2^ = 0.015) (Figure 2b). FATmax in both the EXP group and the CON group did not change significantly (Figure 2c). The VO_2_max was increased significantly (+3.2 mL/kg/min, *p* < 0.01, ES = 0.897), there was no significant change in the VO_2_max in the CON group, and there was a major effect related to the intervention time (F = 7.62, *p* = 0.008, *η*^2^ = 0.020) (Figure 2d). As shown in Figure 3, after seven weeks of intervention, the change in the FO_rest in the EXP group (∆ = 0.59) was significantly higher than that in the CON group (∆ = 0.11) (*p* < 0.05), the change in the MFO in the EXP group (∆ = 0.23) was significantly higher than that in the CON group (∆ = 0.02) (*p* < 0.05), while there was no significant difference in the change of the FATmax between the two groups. The change in the VO_2_max in the EXP group (∆ = 0.12) was significantly higher than that in the CON group (∆ = 0.01) (*p* < 0.05).

### 3.4. Changes in the Fat Oxidation and VO_2_max between genders in the EXP Group

**Figure 4:**
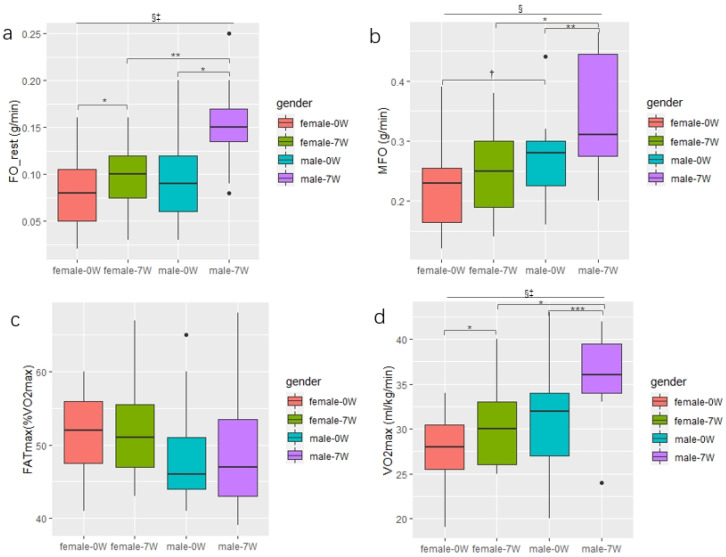
Changes in fat oxidation and VO_2_max between genders in the EXP group. *** denotes a significant difference (*p* < 0.001), ** denotes a significant difference (*p* < 0.01), * denotes a significant difference (*p* < 0.05), ^§^ indicates that there was a primary effect of intervention time (*p* < 0.05), and ^‡^ indicates an interaction between genders and intervention time (*p* < 0.05), a denotes FO_rest of subjects of different genders pre- and post-intervention, b denotes MFO of subjects of different genders pre- and post-intervention, c denotes FATmax of sujects subjects of different genders pre- and post-intervention, d denotes VO_2_max of subjects subjects of different genders pre- and post-intervention.

**Figure 5:**
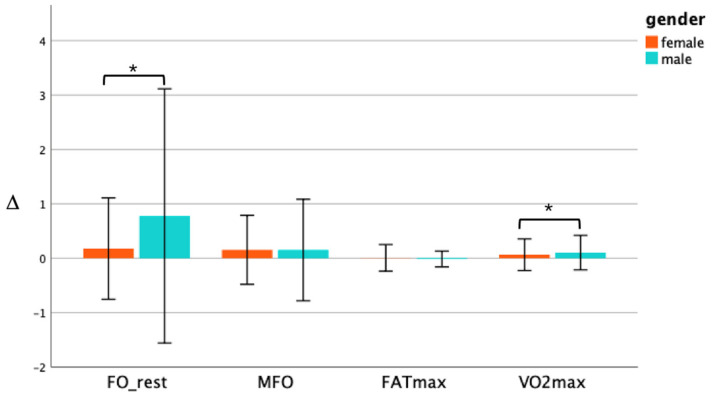
Change percentage in the fat oxidation and VO_2_max between genders in the EXP group. * denotes a significant difference (*p* < 0.05) and ∆ denotes the change pre- and post-intervention.

After seven weeks of CAEH intervention, FO_rest was significantly increased in both the male and female subjects (*p* < 0.05). Males and females increased by 0.05 g/min and 0.02 g/min, respectively, and there was a primary effect of intervention time (F = 19.26, *p* < 0.001, *η*^2^ = 0.143). There was also an interaction between genders and intervention time (F = 6.92, *p* < 0.05, *η*^2^ = 0.051) (Figure 4a). MFO was significantly increased in men after intervention (*p* < 0.01). The value increased by 0.08 g/min in men, and for women the MFO was not significantly increased, but the results tended to be significant (*p* < 0.1), and there was a major effect of intervention time (F = 15.84, *p* < 0.001, *η*^2^ = 0.086) (Figure 4b). The FATmax of both male and female subjects did not change significantly (Figure 4c). The VO_2_max was significantly increased in both male and female subjects (*p* < 0.001, *p* < 0.05), and the VO_2_max of males was increased by 5.0 mL/kg/min, and in females the VO_2_max increased by 2.0 mL/kg/min on average. There was a primary effect of intervention time (F = 31.98, *p* < 0.001, *η*^2^ = 0.102). There was also an interaction effect between genders and intervention time (F = 5.04, *p* < 0.05, *η*^2^ = 0.016) (Figure 4d). As shown in Figure 5, after seven weeks of the CAEH intervention, the change in FO_rest in males (∆ = 0.57) was significantly higher than that in females (∆ = 0.20) (*p* < 0.05), while there was no significant difference in the change of MFO and FATmax between the genders. The percentage change in VO_2_max in male subjects (∆ = 0.16) was significantly higher than that in females (∆ = 0.08) (*p* < 0.05).

## 4. Discussion

Following seven weeks of CAEH, most of the variables of lipid oxidation capacity, body composition, and blood lipids changed in the EXP group; however, there were no changes in the CON group. Regarding the EXP group, BMI, body fat percentage, visceral fat area, and triglyceride levels of the sedentary obesity subjects were significantly reduced, and the FO_rest and VO_2_max increased significantly. In addition, the percentage of changes in the FO_rest and VO_2_max of males was significantly higher than that in females. To the best of our knowledge, this is the first study to examine the effect of CAEH in sedentary obese people.

### 4.1. Body Composition

There were favorable changes in BMI, body fat percentage, and area of visceral fat of the EXP group, but no significant changes in the body weight and skeletal muscle in both groups, pre-and post-intervention.

Exercise training plays an effective role in reducing body fat percentage in adults with overweight or obesity problems, and the changes in body composition in this study agreed with the previous conclusions of a meta-analysis [20] that pointed out that a short- or medium-term duration CAEH intervention can produce fat loss [31]. These results were similarly reported by Cava et al. [32], who found that fat mass decreased and muscle mass remained unchanged. Therefore, when adopting an individual load for this exercise program and considering it as a prescription, CAEH can provide optimal effectiveness for fat reduction. The change in body weight was not significant in this study, and this result corresponds with a recent systematic review. In addition, weight loss was minimal, but reduction in BMI was significantly more effective in aerobic exercise [33]. It was expected that combining HIIT might increase muscle mass and induce visceral fat, however, there was no benefit found in this study of increasing skeletal muscle. Thus, resistance training may be combined in a program for improving skeletal muscle mass in the future.

In addition, the BF percentage and visceral fat area (visceral fat level) of the subjects in the EXP group were significantly reduced, indicating that exercise consumed excess body fat. In particular, visceral fat areas in this study were found to be similar to those found in a study performed by Tan et al. [34] that claimed that individualized FATmax intensity helped to effectively reduce body fat and decrease abdominal fat. The reason for this may be that the progressive load of aerobic exercise relies on fat oxidation, and HIIT exercise increases energy consumption and fat oxidation rate during the recovery period [35]. These findings are similar to those reported in previous meta-analyses that explored the effect of isolated aerobic exercise [36] and combined resistance training [37]. Moreover, future studies may use resistance training as an alternative session to improve muscle growth.

### 4.2. Lipid Profile

One finding from the current study was that, after seven weeks of CAEH intervention, the TC of the EXP group decreased significantly, and there were no significant changes in the other three indicators. However, some studies found that [38] people with good blood lipid levels have more fat energy supply. After the moderate-intensity CAEH intervention, subjects’ blood lipid metabolism indicators significantly improved, and fat oxidation ability also significantly improved. Nevertheless, while the fat oxidation rate increased in this study, except for TC, the other blood lipid metabolism indexes did not change significantly. This may have been due to the fact that most of the subjects’ blood lipid levels were within a normal range, so there were no major fluctuations.

### 4.3. MFO and FATmax

In this study, seven weeks of CAEH intervention significantly improved the FO_rest and MFO of subjects with excessive body fat. However, since an increase in FATmax requires more extended participation in regular exercise, it is recommended that the intervention period of CAEH be extended by more weeks in subsequent trials.

The FO_rest and MFO of subjects in the EXP group increased significantly, but FATmax did not change. This was similar to the results of previous studies that found that MFO generally increased after exercise training [34], while the FATmax generally did not change [39]. Exercise intensity directly affects the transfer of fatty acids and changes in corresponding hormones, and this, in turn, affects the fat oxidation rate [17,18]. The prescribed exercise with a progressive intensity from 40% to 70% in this study had a potentially profound effect on MFO and may be influenced and increased with training [40,41]. During the exercise process to reduce fat, exercise intensity is a crucial factor that affects the rate of fat oxidation. When the exercise intensity changes from moderate intensity to high intensity, the glycolysis system becomes the primary energy supply system. The lactic acid produced by glycolysis will inhibit the entry of long-chain fatty acids into the mitochondria, thereby inhibiting the entry of long-chain fatty acids in the body to utilize fat [42].

It known that MFO and FATmax during exercise are currently considered as indicators of metabolic flexibility [43,44]. Moreover, there is evidence that regular physical activity can improve metabolic flexibility and insulin sensitivity, which is mediated by skeletal muscle contraction activity and triggers a wide range of molecular stimulation and physiological benefits, such as enhancement of mitochondrial activity in addition to an increase in the ability of skeletal muscle to oxidize fatty acids [43]. Compared with untrained people, the oxidation rate and utilization rate of fat are generally higher among trained people [12]. This could be related to the fact that, under a relatively low exercise intensity, muscle fat oxidation increases, glycogen storage slows down, and white adipose tissue lipolysis increases. The increase in MFO induced by training may likely explain the adaptation of fat oxidative decomposition in adipose tissue, and this improves the efficiency of serum non-lipidized fatty acid transport to skeletal muscle and skeletal muscle uptake of fatty acids [45]. In addition to increasing MFO, regular exercise also contributes to the ability to oxidize fat under higher loads [46].

Studies have confirmed that the fat oxidation rate of sedentary obese adults can be increased by aerobic training or interval training [44]. A four-week study found [39] that exercise could not significantly increase FO_rest. This is in contrast to a 12-week study showing that CAEH intervention can increase the fat oxidation rate, both at rest and during exercise [47]. A 12-month aerobic exercise intervention has been shown to significantly increase FATmax [48]. Due to the short duration, this study determined that this was insufficient to elicit maximal fat oxidation.

### 4.4. VO_2_max

VO_2_max is a major index that is used to evaluate the body’s aerobic capacity and cardiorespiratory fitness. The higher the VO_2_max, the stronger the aerobic capacity is, and the more oxygen is absorbed per unit time. Bircher and Knechtle [49] demonstrated the concept of exercise intensity for maximal fat oxidation by comparing sedentary obese adults with athletes. They showed that MFO was highly associated with respiratory capacity, and that increases in MFO paralleled changes in training status.

Considering that fat is one of the primary energy substrates of the human body and its oxidation is entirely dependent on oxygen, aerobic capacity affects fat oxidation capacity [50]. Studies have shown that exercise improves the ability of blood to transport oxygen and the body to absorb oxygen, thus increasing VO_2_max [51]. Obesity, or an overweight status, may not directly lead to a decrease in individual O_2_ uptake, but excess fat may adversely affect oxygen uptake at a sub-maximum intensity [52].

The findings of this study echo the above theory, and the VO_2_max of the subjects in the EXP group was significantly increased after the intervention. This may have been attributed to exercise inducing a higher O_2_ transpiration and mitochondrial O_2_ utilization capacity, partially explaining the greater fat oxidation capacity in endurance athletes [53]. In addition, Weiss et al. [54] demonstrated that exercise effectively reduced body weight (7% weight loss over 16.8 weeks), maintained lean body mass, and increased VO_2_max compared to weight loss achieved through weight loss energy intake restriction alone.

According to the meta-analyses, all training types increased VO_2_max (improved cardiorespiratory fitness, as measured by the maximal oxygen uptake per kg body weight), and the HIIT and aerobic exercise programs were most effective. However, HIIT had a slightly larger effect on VO_2_max than aerobic training. Hence, it is reasonable to suggest that including two types of training can contribute to greater improvements in cardiorespiratory fitness and body composition and enhance overall health. Furthermore, seven weeks of combined exercise can improve VO_2_max, which is more effective in reducing body fat in obese people. The higher the VO_2_max, the higher intensity and the longer the duration of exercise that can be sustained is, increasing energy consumption during exercise, thus burning more fat. Therefore, it is imperative to improve aerobic capacity with appropriate exercise programs to improve fat oxidation and body fat loss.

### 4.5. Gender Differences

Exercise intensity has been shown to be one of the most critical factors that affects substrate utilization [55]. Some studies have compared FO_rest between genders and found that FO_rest in males is higher than in females [56]. In this study, it was found that there was no difference in FO_rest between sedentary Chinese men and women. However, after seven weeks of CAEH intervention, FO_rest was significantly increased in both men and women (Figure 3a), and exercise significantly increased FO_rest in men compared to in women (Figure 4). One analysis showed that MFO was higher in males than in females, and that there was a large effect size (ES = 0.76 ± 0.10). However, female FATmax was higher than in males, but the effect size was smaller (ES = 0.41 ± 0.09) [44]. This was similar to our results, which found that, in sedentary Chinese people who do not exercise, the MFO of males was higher than that of females. However, the results tended to be significant (*p* < 0.1). The FATmax of males was lower than that of females, but not significantly so. These results were consistent with the results of recent extensive cohort studies [57]. The results may also be affected by sample size. Future studies need to accurately calculate sample size to further explore the gender differences in fat oxidation rate caused by exercise.

As observed by Knechtle et al. [22], relative body fat oxidation (i.e., as a percentage of the total energy expenditure) was more significant in women than in men at a steady exercise intensity. During low-intensity exercise, the oxidation rate of carbohydrates and fat in males gradually increased. In contrast, only the fat oxidation rate in females gradually increased, and the oxidation level of carbohydrates remained unchanged [58]. This result indicated that the proportion of fat for energy supply may be higher in females. However, the oxidation rate of fat was lower than in males. In a study on gender and FATmax, it was found that the proportion of fat energy supply in males and premenopausal women was significantly higher than that in males under medium and high exercise intensity [59]. In addition, similar conclusions were also drawn in related studies, and this result may have been due to the influence of estrogen levels in females [60]. Therefore, gender differences in FATmax still require further study.

In addition, there was a significant positive correlation between VO_2_max and MFO [57]. In this study, both VO_2_max and fat oxidation rates increased significantly after the intervention. Moreover, by comparing the percentage changes of the VO_2_max of males and females in the EXP group after the intervention, it was found that the VO_2_max of males increased more. This may have been because the body’s ability to use oxygen and the mitochondrial function of males were better than those of females after the CAEH intervention.

### 4.6. Limitations

Some limitations of this study included the fact that (I) this study was a non-randomized controlled trial, which may have led to allocation bias, but fortunately there were no significant differences in the baseline between groups; however, their confounding effects can never be eliminated, therefore, further studies will need to explore the randomized controlled trial. (II) Although the sample size was not enough in the end, we calculated the effect size in order to accurately interpret the results. (III) The progressive aerobic exercise and HIIT were not studied separately. Hence, it was not possible to compare the effect difference between single exercise and CEAH. (IV) Only dietary education was conducted for the subjects, and dietary intervention was not conducted. Therefore, different dietary habits may have affected the results.

## 5. Conclusions

Seven weeks of CAEH intervention effectively increased FO_rest, MFO, and VO_2_max in sedentary Chinese people with excess fat percentages, and the improvement in FO_rest and VO_2_max were more pronounced in males than in females. In addition, seven weeks of CAEH also improved body composition and TC levels in sedentary Chinese people with excess fat percentages. Future studies could combine resistance training to promote muscle gains and improve fat oxidation.

## Figures and Tables

**Table 1:** Basic characteristics of the participants.

Variables	CON Group (n = 25)	EXP Group (n = 31)
**Basic information**		
Age (years)	40.1 ± 6.9	41.2 ± 8.9
Male, n (%)	11 (44%)	13 (42%)
Height (cm)	166 ± 8.0	166 ± 9.2
**Fat oxidation capacity**		
FO_rest (g/min)	0.09 ± 0.03	0.09 ± 0.04
MFO (g/min)	0.25 ± 0.08	0.24 ± 0.08
FATmax (%VO_2_ max)	49 ± 7	50 ± 6
**Aerobic capacity**		
VO_2_max (ml/kg/min)	30.3 ± 5.5	29.1 ± 5.1
**Body composition**		
BMI (kg/m^2^)	25.2 ± 2.0	26.4 ± 4.1
Weight (kg)	69.2 ± 10.2	72.2 ± 16.4
Body fat percent (%)	32.3 ± 5.1	32.8 ± 4.8
VFA (cm^2^)	97.5 ± 22.7	103.2 ± 35.3
Skeletal muscle (kg)	27.3 ± 6.3	27.0 ± 7.3
**Lipid profile**		
TC (mmol/L)	4.8 ± 0.9	5.1 ± 0.8
TG (mmol/L)	1.6 ± 1.0	1.5 ± 1.0
HDL-C (mmol/L)	1.5 ± 0.3	1.6 ± 0.4
LDL-C (mmol/L)	3.0 ± 0.9	3.5 ± 0.7

Note: Data are means ± SD, all variables are statistically without difference, CON group: control group, EXP group: experimental group, FO-rest: resting fat oxidation rate, MFO: maximal fat oxidation, BMI: body mass index, VFA: visceral fat area, TC: total cholesterol, TG: triglycerides, HDL-C: high-density lipoprotein cholesterol, LDL-C: low-density lipoprotein cholesterol.

**Table 2:** Change in body composition pre- and post-intervention.

	CON Group (n = 25)			EXP Group (n = 31)		
	Pre	Post	∆	Pre	Post	∆
BMI (kg/m^2^) ^∮^	25.2 ± 2.0	25.1 ± 2.4	−0.01 ± 0.22	26.4 ± 4.1	25.2 ± 3.7 **	−0.06 ± 0.18 ^b^
Weight (kg)	69.2 ± 10.2	67.9 ± 17.1	−0.09 ± 0.30	72.2 ± 16.4	68.0 ± 19.8	−0.09 ± 0.23
Body fat percent (%) ^§^	32.3 ± 5.1	31.3 ± 5.4	−0.07 ± 0.23	32.8 ± 4.8	29.6 ± 5.4 ***	−0.13 ± 0.18 ^a^
VFA (cm^2^) ^§^	97.5 ± 22.7	95.3 ± 26.3	−0.04 ± 0.30	103.2 ± 35.3	90.4 ± 31.7 ***	−0.14 ± 0.20 ^a^
Skeletal muscle (kg)	27.3 ± 6.3	27.2 ± 6.3	−0.02 ± 0.10	27.0 ± 7.3	27.7 ± 7.4	0.01 ± 0.20
TC (mmol/L) ^§^	4.8 ± 0.9	4.7 ± 0.9	−0.01 ± 0.09	5.1 ± 0.8	4.7 ± 0.8 *	−0.05 ± 0.16 ^a^
TG (mmol/L)	1.6 ± 1.0	1.6 ± 1.5	0.01 ± 0.34	1.5 ± 1.0	1.4 ± 0.8	−0.02 ± 0.28
HDL-C (mmol/L)	1.5 ± 0.3	1.5 ± 0.3	−0.01 ± 0.09	1.6 ± 0.4	1.6 ± 0.4	0.02 ± 0.14
LDL-C (mmol/L)	3.0 ± 0.9	2.9 ± 0.9	−0.02 ± 0.17	3.5 ± 0.7	3.2 ± 0.8	−0.03 ± 0.21

Note: Data are means ± SD. * denotes a significant change from pre-intervention *(p <* 0.05), ** denotes a significant change from pre-intervention *(p <* 0.01), *** denotes a significant change from pre-intervention (*p* < 0.001), ^§^ denotes a primary effect of the intervention time (*p* < 0.05), and ^∮^ denotes the interaction effect between the CAEH and the intervention time (*p* < 0.05). ∆ denotes change between pre- and post-intervention. ^a^ denotes ∆ value of EXP group was significantly different from that of the CON group, *p* < 0.05, ^b^ denotes ∆ value of EXP group was significantly different from that of the CON group, *p* < 0.1.

## Data Availability

The data presented in this study are available on request from the corresponding author.
